# Amyloid–Gold Nanoparticle Hybrids for Biocompatible Memristive Devices

**DOI:** 10.3390/ma16051884

**Published:** 2023-02-24

**Authors:** Aoze Han, Liwei Zhang, Miaocheng Zhang, Cheng Liu, Rongrong Wu, Yixin Wei, Ronghui Dan, Xingyu Chen, Ertao Hu, Yerong Zhang, Yi Tong, Lei Liu

**Affiliations:** 1 College of Integrated Circuit Science and Engineering, Nanjing University of Posts and Telecommunications, Nanjing 210023, China; 2 Institute for Advanced Materials, Jiangsu University, Zhenjiang 212013, China; 3 College of Electronic and Optical Engineering & College of Flexible Electronics (Future Technology), Nanjing University of Posts and Telecommunications, Nanjing 210023, China

**Keywords:** amyloid–gold hybrids, memristor, ion migration, brain inspired, boolean logic

## Abstract

Biomolecular materials offer tremendous potential for the development of memristive devices due to their low cost of production, environmental friendliness, and, most notably, biocompatibility. Herein, biocompatible memristive devices based on amyloid–gold nanoparticle hybrids have been investigated. These memristors demonstrate excellent electrical performance, featuring an ultrahigh *R_off_/R_on_* ratio (>10^7^), a low switching voltage (<0.8 V), and reliable reproducibility. Additionally, the reversible transition from threshold switching to resistive switching mode was achieved in this work. The arrangement of peptides in amyloid fibrils endows the surface polarity and phenylalanine packing, which provides channels for the migration of Ag ions in the memristors. By modulating voltage pulse signals, the study successfully imitates the synaptic behavior of excitatory postsynaptic current (EPSC), paired-pulse facilitation (PPF), and the transition from short-term plasticity (STP) to long-term plasticity (LTP). More interestingly, Boolean logic standard cells were designed and simulated using the memristive devices. The fundamental and experimental results of this study thus offer insights into the utilization of biomolecular materials for advanced memristive devices.

## 1. Introduction

Brain-inspired computing, operating in the same manner as the human brain, has evolved as a new computing paradigm that circumvents the von-Neumann bottleneck [1,2]. The implementation of artificial synapses and logic-in-memory using electronic devices is considered the key step for brain-inspired computing architecture. Memristors have been demonstrated to be promising devices with vast application potential for their inherent benefits of low power consumption, multilevel data storage, etc. [3,4,5,6]. However, the reported memristive devices are more inorganic materials as the functional layer. Many biocompatible devices based on biomaterials have been explored and fabricated for biological sensing and detection [7,8,9,10], for their inherent renewability, biocompatibility, ecofriendly qualities, and biodegradability. Also, natural biomaterials are more suitable for the biorelated hardware realization of brain-inspired computing [11,12,13,14]. In addition, their application opportunities in implantable computing and human–machine integration surpass those of conventional inorganic components. Therefore, it is of great significance to develop high performance biomolecule-based memristive devices.

Recently, researchers proposed ferritin-based protein active layers with metal ions as well as fibroin or sericin-based protein active layers without metal ions. The protein-based resistive-switching memory devices present a high on/off ratio of 10^6^ with a long retention time, which is necessary and desirable for future biointegrated systems [15,16,17,18]. Furthermore, protein nanowires harvested from the bacterium Geobacter sulfurreducens were used as an active layer to fabricate a type of diffusive memristor with biological voltages of 40–100 mV, achieving the biological amplitude [14]. However, the exact requirements of an active layer in biomolecule-based memristors were not explored clearly. Natural polymers, i.e., fibroin or sericin as the mixture could not give the exact role due to the impurity. Meanwhile, the pilin-based protein nanowires from bacteria are assembled from big proteins with hundreds of amino acids, which displayed the complexity of understanding the effect of protein on biomolecule-based memristors. In comparison, peptides exhibited the advantages of structural programmability and versatile functionality by a rational sequence design, which could guide self assembly and control the variety of properties, i.e., electro-active property, molecular binding, and inorganic material growth [19,20,21,22]. Previously, we have identified that amyloid peptide (SNNFGAILSS, hIAPP_20–29_), ranging from 20 to 29 of human islet amylin polypeptide (hIAPP_1–37_), was able to self-assemble into β-sheet rich amyloid fibrils [23]. It has been also found that amyloid peptide nanostructures with the specific sequence leading to the dipole moment or formation of quantum confined domains [24,25,26,27], endowing the peptides with desired electronic properties for photonic materials, temperature-dependent electrically conductive materials, ferroelectric materials, etc. Amyloid-based materials could possibly meet the requirement as the active layer of memristor, however, they have rarely been explored before with a clear mechanism. Therefore, it is necessary to investigate the possibility of amyloid-based fibrils and gold nanoparticles reduced on the amyloid fibrils as the active layer of memristive devices, due to the advantages of structural simplicity and versatile properties.

In this work, the biocompatible memristive devices based on amyloid fibrils (hIAPP_20–29_ self-assembled fibrils) and Au–fibril hybrids were fabricated and systematically measured, further exhibiting the superiority of brain-inspired computing and Boolean logic applications. The introduction of metal particles may facilitate the stable growth of conductive filaments, enhancing the performance of memristors [28,29,30]. The switching mode of the Au–fibril hybrid memristive devices can be determined by the compliance current (CC). The Au–fibrils hybrid-based memristor showed superior performance compared to the conventional devices [31,32,33]. Moreover, the conductive mechanism of Au–fibrils hybrid-based memristors can be explained by the space charge limited current (SCLC) model. Additionally, synaptic functions are emulated successfully by Au–fibril hybrid-based memristive devices. More interestingly, on the basis of the nonvolatile model of the memristive devices, two types of Boolean logic have been implemented. This work provides a new candidate for biocompatible memristive devices for brain-inspired computing applications, etc.

## 2. Materials and Methods

### 2.1. Reagents and Chemicals

Chloroauric acid (HAuCl_4_·4H_2_O) and sodium borohydride were purchased from Sigma-Aldrich. All chemical reagents were analytical grade and used without further processing. The hIAPP_20–29_ (SNNFGAILSS) was synthesized and analyzed by Guoping Pharmaceutical Co., Ltd. (Hefei, China). Deionized water with a resistivity of 18.2 MΩ was used for all solution preparation.

### 2.2. Construction and Characterization of hIAPP_20–29_ Fibrils

The polypeptide hIAPP_20–29_ was dissolved in Milli-Q water at a concentration of 400 μM. The mixture was sonicated for 10 s to ensure full dissolution. Subsequently, the solution was heated in a thermo-shaker (PHMT, Grant Instruments, Royston, UK) at stable conditions and 37 °C for 12 h. In the end, the resulting suspension was stored at 4 °C for the following test. Circular dichroism (CD) spectra of the resulting polypeptide solution were performed with a scan speed of 50 nm/min and a slit width of 2 nm on a JASCO PTC-348W1 spectropolarimeter. All samples were measured three times. The atomic force microscope (AFM) images of polypeptide solution with a resolution of 512 × 512 pixels were completed on Multimode VIII SPM (Bruker, Berlin, Germany) in tapping mode in air, in a quiet room at room temperature.

### 2.3. Synthesis and Characterization of the Au-Fibrils Hybrids

The hIAPP_20–29_ fibrils templated Au particles were fulfilled by the reduction of Au^3+^ with the hIAPP_20–29_ fibrils as templates and the NaBH_4_ as reductant. Typically, 800 μL of hIAPP_20–29_ fibrils solution (400 μM) were mixed with 800 μL of 2 mM HAuCl4 aqueous solution under stirring for 15 min. Next, 800 μL of 10 mM freshly prepared ice-cold NaBH4 aqueous solution were added to the mixed solution. The Au–fibrils hybrids were obtained after 10 min. The morphology of the Au–fibrils hybrids was performed with an atomic force microscope and a transmission electron microscope (FEI, model CM12, NL, Lausanne, Switzerland) with energy dispersive spectroscopy elemental mapping. UV-Vis spectroscopic characterization of the Au–fibrils nanocomposite solutions was achieved on a UV2600i spectrometer (Shimadzu, Kyoto, Tokyo, Japan). X-ray photoelectron spectra (XPS) of the representative Au nanoparticles were recorded on an ESCALAB 250 spectrometer (PHI5000 Versa Probe) using Al Kα radiation at 1486.6 eV. The resolution of the XPS is ±0.1 eV. The binding energies of the Au–fibril hybrids were analyzed with respect to the C 1s peak of contaminated carbon at 284.6 eV. X-ray diffraction (XRD) was put in (Rigaku D/max-rA) to identify the crystalline-phase structures of the Au–fibril hybrids. Circular dichroism (CD) spectra of the Au–fibril hybrids were tested as mentioned above. The structural model, the hydrophilicity, hydrophobicity, and the molecular electrostatic potential (MEP) surface analysis of the hIAPP_20–29_ fibrils were presented using PyMol. The structural model of hIAPP_20–29_ self-assembled filament was quoted from our previous work [23].

### 2.4. Fabrication of Memristive Devices

The crossbar of the Au–fibril hybrids-based memristors and amyloid fibril-based memristors were fabricated on the Pen substrates. The flexible Pen sheets (20 cm × 15 cm) were ultrasonically cleaned with absolute ethanol and cropped into a rectangular substrate with a length of 2 inches. The bottom electrode of Pt (100 nm, 25 °C, 17 min) was deposited on the obtained Pen substrates by physical vapor deposition assisting with magnetron sputtering with a shadow mask. After the process of the bottom electrode, the resistive-switching layer of the Au–fibril hybrids were prepared by drop-coating with a shadow mask and naturally dried (25 °C, 1 h) in the ambient environment. The deposition of the amyloid fibrils was by the same experimental steps. Finally, the top electrode of Ag (100 nm, 25 °C, 7 min) was deposited by similar experimental conditions with Pt. The electrical performance of the Ag/amyloid fibrils/Pt and Ag/Au-fibrils hybrids/Pt memristive devices were measured and evaluated by the Keithley 4200A-SCS semiconductor analyzer and Cascade Micromesh MPS150. The electric properties of the prepared amyloid fibrils and Au–fibril hybrids were analyzed by ohmic conduction of the membrane with a metallic probe (W). During the electrical measurements of the mentioned memristors, the Ag top electrode was applied by a positive voltage signal of the source measure unit (SMU), and the Pt bottom electrode was grounded.

## 3. Results and Discussion

### 3.1. Material Selection and Characterizations

**Figure 1:**
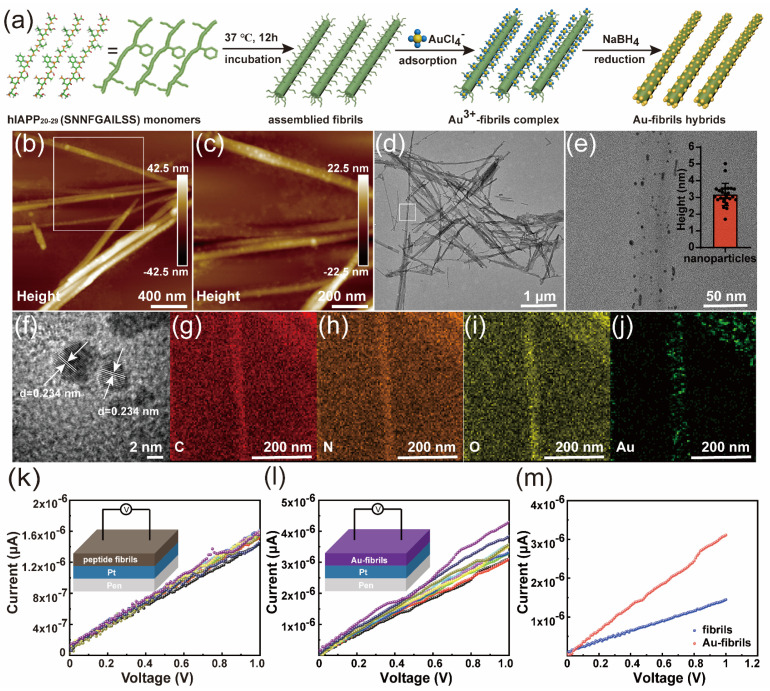
Constructions and characterizations of the gold particle-fibrils (Au-fibrils) hybrids. (**a**) Schematics of constructing amyloid fibrils and Au-fibrils hybrids; (**b**,**c**) AFM images of the Au–fibril hybrids; (**d**,**e**) TEM images of the Au–fibril hybrids, the inserts are the corresponding gold particle size distribution; (**f**) HRTEM image of gold particles of (**e**). The interplanar spacing is about 0.234 nm; (**g**–**j**) Elemental mapping (C, N, O, Au) of the Au–fibril hybrids of (**e**); (**k**–**m**) The electro-property of amyloid fibril and Au–fibrils. I-V curve of amyloid fibrils and Au–fibrils characterized by setting device; (**k**) I-V curves of amyloid fibrils for multiply tests; (**l**) I-V curves of Au–fibrils for multiply tests; (**m**) the comparison of I-V curves between amyloid fibrils and Au–fibrils.

Initially, schematic illustrations of self-assembled amyloid fibrils and Au–fibril hybrids via a conventional wet-chemistry reduction process were displayed. As expected, hIAPP_20–29_ could self assemble into nanofibrils with a height of 40.46 ± 15 nm (Figure S1a,b), characterized by AFM and TEM. CD spectra could further determine the secondary structure of the peptide to be a β-sheet in nanofibrils (Figure S1c). Subsequently, it has been confirmed that hIAPP_20–29_ assemblies could facilitate the synthesis of metallic Au nanostructures, with the aid of sodium borohydride (NaBH_4_) as the reductant for small-sized and spherical particles. The Au–fibril hybrids were prepared successfully (Figure 1a and Figure S2a). The spherical Au NPs were observed with a particle size of 3.17 nm and well anchored and dispersed on the surfaces of amyloid nanofibrils (Figure 1b–e), which could not affect the secondary structure of the peptide (Figure S2b). The HRTEM image shows the lattice fringe spacings of about 0.234 nm, assigning to the Au (111) crystal plane of the face-centered cubic (fcc) structure of metallic Au^0^ (Figure 1f). According to the survey of the XPS spectrum, the obvious peak of Au 4f can be observed and a zero valence state (Au^0^) at 87.38 eV and 83.88 eV in the sample was detected (Figure S2c). The XRD pattern with a major single (111) peak along the full scanning angle range was displayed and it was demonstrated that the AuCl_4_-ions were reduced to the zero valence state (Figure S2d). Meanwhile, the energy-dispersive X-ray spectroscopy mapping characterization of Au–fibril hybrids revealed that C, N, O, and Au elements were the main ingredients of the fibrils (Figure 1g–j). The electric properties of amyloid fibrils and Au-fibrils hybrids were explored, which is the key to further evaluating the performance of biocompatible memristive devices. Figure 1k,l showed the linear I-V curves of the amyloid fibrils and the Au–fibril hybrids in the order of 10^−6^ μA, respectively. In comparison, it was clearly observed from Figure 1m that the conductivity of Au–fibrils was significantly enhanced by the hybridization of Au particles, which may contribute to the improvement in switching characteristics of memristive devices.

### 3.2. Device Structures and Electrical Properties

**Figure 2:**
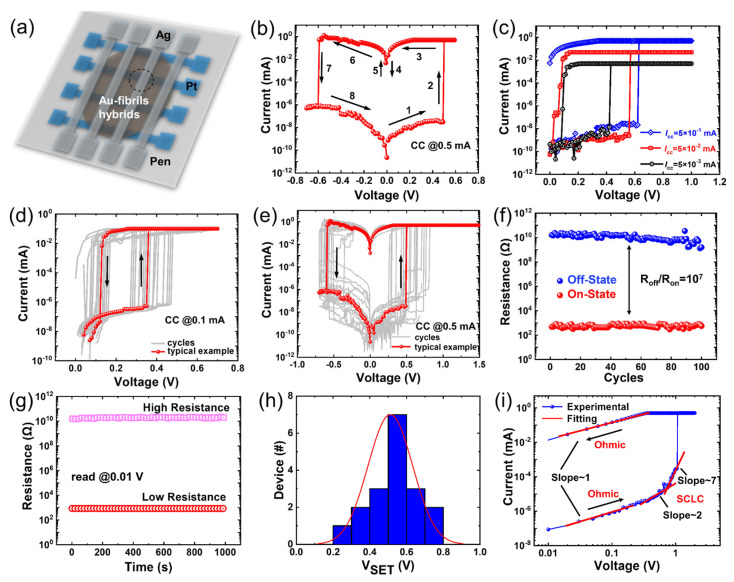
Biocompatible devices based on the Au–fibril hybrids and electrical characterization. (**a**) Schematic of memristor crossbar array; (**b**) Typical current-voltage (I-V) characteristics of Au–fibril hybrids-based memristive devices. The resistance switches from the HRS to LRS through a SET process (1–3), and the state can be reversed via a RESET process (4–7); (**c**) The current-voltage (I-V) characteristics of the SET process under different current compliances set from 5 μA to 0.5 mA; (**d**) Threshold switching I-V characteristic of the devices under current compliances of 0.1 mA; (**e**) Resistive switching I-V characteristic of the devices under current compliances of 0.5 mA; (**f**) The endurance of off-state and on-state of the devices for 100 cycles (RS mode). The switching ratio of *R_off_/R_on_* is about 10^7^; (**g**) The retention performance (~10^3^s) of both high and low resistance states (RS mode); (**h**) Statistics of the SET voltage from 18 devices (RS mode); (**i**) The curves and fitting results of the positive region in the log-log scale (SET process in RS mode).

Subsequently, we fabricated the biocompatible memristive devices based on the amyloid fibrils or Au–fibril hybrids as the active layers and tested the performance. In the case of amyloid fibrils, to investigate the effects of programming current on the switching behavior of amyloid fibrils-based memristive devices (demonstrated in Figure 2a), positive current-voltage curves with different CCs (0.01 μA to 0.5 μA) are plotted (Figure S3a). It can be observed clearly that the response current of the device is not able to reach the CC (0.5 μA) at the moment of the resistive switching. It retained threshold switching (TS) behavior, indicating that the switching mode of the device is generally independent of CC. The typical TS I−V curves of amyloid fibrils-based memristive devices were obtained with thirty cycles (Figure S3b). However, Au–fibril hybrid-based memristive devices exhibited distinct performance (Figure 2b). During the positive voltage sweep (0 V to 0.6 V), the devices switched from a high resistance state (HRS) to a low resistance state (LRS), which is defined as the SET process. Under the RESET process of negative voltage sweep (0 V to −0.7 V), the device recovers to HRS. To investigate the effects of programming current on the switching behavior of the devices, positive current-voltage curves with different CCs (1 μA to 0.1 mA) are plotted in Figure 2c. It can be observed that the switching voltages exhibit a small variation (0.5 V to 0.8 V), indicating that it is generally independent of CC. However, the switching mode of the device is changed from volatile TS behavior under low CC (<0.1 mA) to nonvolatile RS behavior under higher CC (>0.5 mA). The typical I-V curves of TS mode are illustrated in Figure 2d. During the backward voltage sweep (0.7 V to 0 V), the devices spontaneously switch from LRS to HRS, which may be attributed to the automatic rupture of Ag CFs [34], revealing that the devices are volatile (CC: 0.1 mA). Cycles of I-V curves of the RS mode under higher (CC: 0.5 mA) are also plotted in Figure 2e. When the forward voltage was reversely scanned from 1.5 V to 0 V, the memristor remained in a low resistance state, which means the devices are nonvolatile. Thirty consecutive voltage sweeps were applied to the Ag–Au–fibrils hybrids and the Pt memristor devices to evaluate the endurance performance (Figure 2f). The cumulative distribution of SET voltage (V_SET_) and RESET voltage (V_RESET_) for 30 continuous cycles was calculated in Figure S4, implying the range of V_SET_ from 0.2 V to 0.8 V and the V_RESET_ from −0.6 V to −0.4 V, respectively. The switching ratio (R_off_/R_on_) is about 10^7^, which meets the requirements for nonvolatile memory [35]. The high switching ratio is attributed to the complete growth of the silver conductive filaments under the influence of the low electric potential field of the Au nanoparticles. The HRS (off-state) and LRS (on-state) can stably maintain for 1000 s under a low read voltage of 0.01 V (Figure 2g). The switching voltage of devices exhibits the rule of a normal distribution from the measured results of eighteen random devices (Figure 2h), implying acceptable device-to-device uniformity. The switching voltage is derived from the average value of 10 cycles for each measured device.

Table 1 summarized the switching characteristic of the recently reported hybrid memristor. Compared to other hybrid memristive devices, the Au–fibril devices have better switching performance, such as low switching voltage, high switching ratio, and two controllable and stable switching modes. This is highly important for practical applications.

### 3.3. Conductive Mechanism Investigations

**Figure 3:**
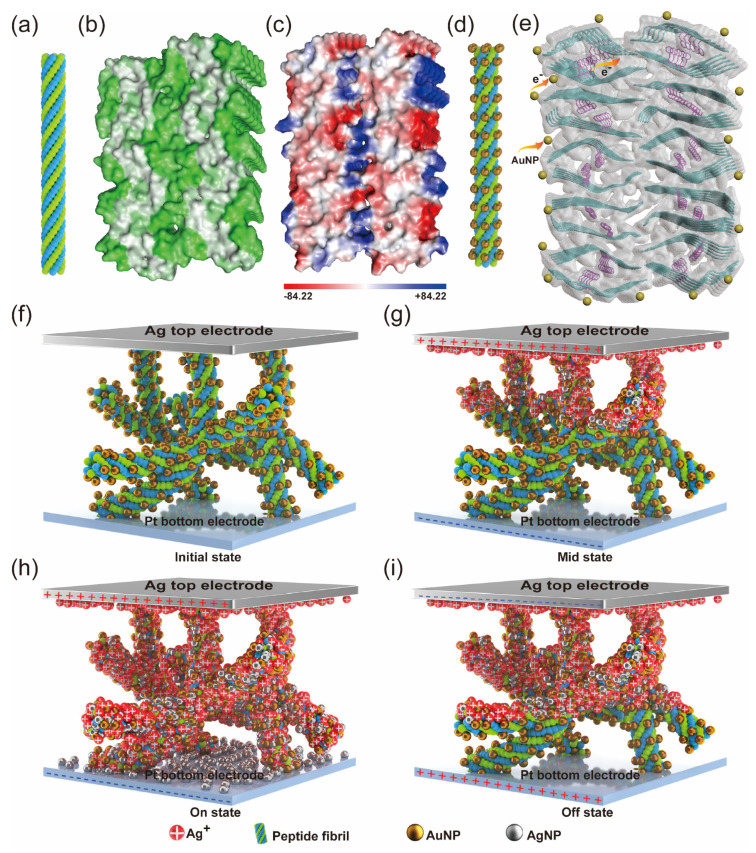
Resistance switching mechanism. (**a**–**e**) The schematic and structural model of amyloid peptide filament quoted from our previous work [23]. (**a**) The schematic model; (**b**) The model of amyloid filament exhibiting the hydrophobic (grey color) and hydrophilic areas (green color) simulated by PyMol; (**c**) The distribution of positive charge (blue color) and negative charge interface (red color) on the amyloid filament simulated by PyMol. The unit is kcal/mol; (**d**) The schematic model of Au–fibril hybrids; (**e**) The model of Au–fibril hybrid displaying the possible pathways of charge or electron on the surface of the fibril due to the polarity, or inside of fibril due to the packing of phenylalanine; (**f**–**i**) Schematics of the conductive mechanism of Ag/Au-fibril hybrids/Pt memristive device. “+” represents positive charge, “−” represents negative charge; (**f**) AuNPs distributed in Au–fibril hybrids layer. HRS at this time; (**g**) Ag atoms aggregated around AuNPs under the action of the electric field from Ag to Pt; (**h**) a conductive filament was formed by Ag atoms between Ag and Pt electrodes. LRS at this time; (**i**) The destruction of the conductive filament by the negative voltage bias.

In order to investigate the conductive mechanism of the Ag/Au–fibril hybrids/Pt memristors, the nonlinear fitting and analysis of the I-V curves (positive region) are carried out (Figure 2i), which are plotted on a double-log scale. It can be observed that when the applied voltage is at a low level, the current exhibits a linear relationship with the voltage, which obeys the Ohmic conduction behavior (slope ~ 1) related to mobile electrons from thermal excitation. As the voltage increased, the relationship followed the Child’s Law conduction (slope ~ 2) [42]. When the voltage is raised around 0.8 V, the current is proportional to the elegant nth power (*n* ~ 7), which may be ascribed to the formation of Ag conductive filaments (CFs) and injected charges distribution of the traps [43]. Consequently, the resistance-switching mechanism agrees with the trap-controlled SCLC model [44]. The schematic diagrams of the possible conductive mechanisms of the Ag/Au-fibril hybrids/Pt memristive devices are further discussed (Figure 3). The vertical top electrodes and the horizontal bottom electrodes are arranged crosswise, and the hybrids were deposited in the middle of two electrodes as the switching layer. The conductive filaments (CFs) of the devices might be formed along the location of the Au–fibril hybrids, which is mainly due to the formation of the conductive pathway (electron hopping paths), however, they are different from the reported metallic conduction of Ag CFs [45,46]. Moreover, the structural analysis of amyloid fibril and Au–fibril hybrids is significant for understanding the formation of the conductive pathway. The structural model of peptide fibrils was explored by Cryo-EM in our previous work. Based on the structural information, we constructed the schematic and structural model of the amyloid peptide filament by molecular dynamics simulation (Figure 3a,b). The surface polarity of the peptide filament was displayed, providing the possible ways of charge and electron transfer (Figure 3c). Au–fibril hybrids showed a similar structural model of peptide filament, however, with the hybridization of gold nanoparticles (Figure 3d). In this case, the charge or electron could not only hop along the surface of the filament but also follow the path inside of the filament built by phenylalanine packing (Figure 3e). The observation of a possible conductive pathway in amyloid fibrils and Au–fibril hybrids has not been achieved before, which provides a more clear insight into the conductive mechanism of biomolecule-based memristors. Based on the experimental results and the structural model proposed for the amyloid filament, a working mechanism was exhibited (Figure 3f–i). Fibrils and the Au–fibril matrix were formed in the active layer, which is crucial for the good performance of the memristor. The distribution and arrangement of fibrils could provide effective ionic pathways for Ag^+^ transport when the voltage is applied. The general electro-migration process is consistent with the previous report about protein-based memristors and the number of electron hopping paths in the active layer is closely related to the LRS and HRS, which could be modulated by the applied voltage and compliance current. Differently, in this case, the migration of Ag CFs in Au–fibril-based memristors may be facilitated by the attraction of Au–fibrils surrounded with the lower potential wells [47,48]. To be more specific, when the positive voltage was applied to the Ag electrode, the Ag was oxidized into Ag ions. At the same time, due to the lower potential around the Au-fibrils, the Ag ions were adsorbed around them. As schematically demonstrated in Figure 3g, when the positive bias was applied to the device, the Ag ions can be attracted by these wells, followed by the reduction of Ag atoms. The CFs were formed from Ag atoms reduced by electrons emitted from the negatively biased Pt electrode, resulting in the transition from HRS to LRS (Figure 3h). When a positive voltage was applied to the Pt electrode, the CFs were destroyed due to the oxidation of Ag atoms near the Pt electrode, and the device switched to HRS (Figure 3i). As a result, the performance of Au–fibril-based memristors, including switching ratio and stability, has been enhanced due to adequate and stable growth of Ag CFs. In addition, dual switching modes, i.e., RS and TS, have been realized in Au–fibril- based memristors, rather than a single switching mode in the devices without Au–fibrils [14,49,50], which is important for the implementation of neuromorphic computing.

### 3.4. Brain-Inspired Applications

**Figure 4:**
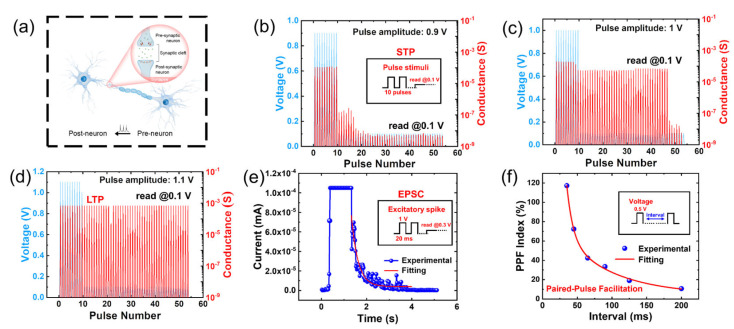
Brain-inspired simulation using the Au–fibril hybrids-based memristive devices. (**a**) The structure of biological synapses, including the pre- and postneuron parts; (**b**–**d**) Responding conductance at 0.9 V–1.1 V pulse amplitude. The transition from STP to LTP; (**e**) The simulated EPSC behavior; (**f**) The simulation of PPF behaviors of a biological synapse.

After the exploration of the conductive mechanism of amyloid fibrils or Au–fibril hybrids-based memristor, the emulation of various synaptic functions based on the constructed memristors was performed. In neuroscience, synaptic plasticity of neurons (Figure 4a), the biological basis of learning and memory in the brain, refers to the property that the strength of synaptic connections (synaptic weight) can be adjusted [51]. Synaptic plasticity is divided into short-term plasticity (STP) and long-term plasticity (LTP). Weak stimulus reaching the presynaptic membrane results in the release of neurotransmitters, thus enhancing synaptic connections. However, this phenomenon lasts only a few milliseconds, which is called STP. The duration of the synaptic transition can be extended by a strong stimulus, which occurs in LTP. In this section, the synaptic behavior of the transition from STP to LTP can be mimicked by tuning the input of voltage pulse to the Ag/Au-fibril hybrids/Pt memristive devices. In our memristive devices system, the top electrode is regarded as the presynapse and the bottom electrode is as the postsynapse. The Ag ions act as neurotransmitters to alter synaptic weight, which is demonstrated in Figure 4b–d. The STP can be realized by voltage pulses with weak amplitude (0.9 V) to the devices (Figure 4b). The conductance in response decayed after the stimulation pulses are withdrawn, which is accounted for in the automatic rupture of Ag CFs. With the increasing pulse amplitude (1 V), the conductance of the devices exhibits a longer duration compared to previous stimulation (Figure 4c). The results suggest that the enhanced stimuli can strengthen the synaptic connections and promote the transition from STP to LTP. When pulses with strong amplitude (1.1 V) were applied to the device, they can be sustained in high conductance states due to the strong and enhanced CFs (Figure 4d), indicating the realization of LTP. The excitatory postsynaptic current (EPSC) is generated after the postsynaptic membrane received the excitatory neurotransmitters released by the presynaptic cell, which is a common phenomenon in neural science [52]. The EPSC process was simulated successfully based on Ag/Au-fibril hybrids/Pt memristive devices with continuous pulse stimuli (1 V, 50 HZ), as illustrated in Figure 4e. To investigate the time-dependent learning mechanism of the devices, the descriptive fitting function of the EPSC process is provided [53,54]

(1) $$I\left(t\right)=I_{0}+A\;\exp\left(-t/\tau \right)$$

where $I\left(t\right)$ is the time-dependent current response, $I_{0}$ is the current at the stable level, A and τ are factor item and relaxation time, respectively. The fitting results exhibited the value of τ in the EPSC process is 297 ms (Figure 4e), which is related to the response of biosynapses [55]. Moreover, paired-pulse facilitation (PPF) is a crucial synaptic function in biological synapses that plays a significant role in information processing [56]. PPF reveals that synaptic weight can be enhanced by two consecutive pulses, however, the effect of this enhancement is weakened with increasing intervals between the pulses [57]. Figure 4f illustrates the simulated PPF results of Ag/Au-fibril hybrids/Pt memristive devices, which are fitted with an exponential function:

(2) $$\mathrm{PPF}\;\mathrm{index}\;=\frac{\left(I_{2}-I_{1}\right)}{I_{1}}\times 100\%=A_{1}\exp\left(-t/\tau_{1}\right)+A_{2}\exp\left(-t/\tau_{2}\right)$$

Here, $I_{1}$ and $I_{2}$ are the current responses of the devices after the first and second pulses. The decaying terms $\tau_{1}$ and $\tau_{2}$ have values of 12 ms and 72 ms, respectively, which align with biological functions [58]. The emulated PPF behavior of the as-constructed devices is attributed to the increasing Ag CFs under high-frequency successive pulses. The simulation of PPF using Au–fibril hybrids-based memristors could potentially advance the development of artificial synapses for neuromorphic computing.

### 3.5. Boolean Logic Construction

**Figure 5:**
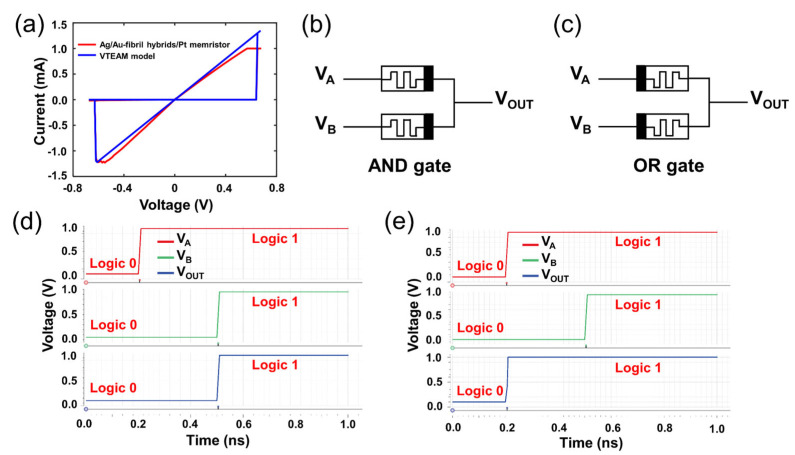
Boolean logic application. (**a**) The I-V curves and model fitting results of memristive device; (**b**,**c**) The circuit of AND gate and OR gate, black represents the top electrode; (**d**,**e**) Logic circuit simulation results.

To establish the viability for circuit implementations, the digital circuit modules need to be realized to connect the biosynaptic devices through neuromorphic computing. Boolean logic is the cornerstone of digital circuit operations. In this study, the logic AND and the logic OR have been realized by using the Au–fibril hybrids-based memristive devices, as shown in Figure 5. The I-V curves were fitted by the VTEAM model in MATLAB (Figure 5a) [59], from which the fitting curves possess similar switching voltage and resistance states with experimental data. The fitting results have been utilized for circuit simulation in Verilog A of virtuoso. According to the memristor ratioed logic in the previous work [60], the implementation of Boolean logic was closely related to the voltage distribution of the memristive devices. Figure 5b,c indicate the circuits of AND gate and OR gate. The logic “0” was represented by a low voltage (0.1 V) while the logic “1” was represented by a high voltage (1 V). Figure 5d,e exhibit the simulation results of the logic AND and the logic OR. The results indicate that Au–fibril hybrids-based memristors present the potential in a Boolean logic application, and are also beneficial for further biocompatible digital circuit design.

## 4. Conclusions

In summary, biocompatible materials of amyloid fibrils and Au–fibril hybrids have been used as the functional layer of memristors for the first time. The amyloid fibrils-based memristors exhibited the volatile threshold switching (TS) behavior, while the switching mode of the Au–fibril hybrids memristive devices can be determined by the compliance current (CC), with volatile threshold switching (TS) behavior at low CC (0.1 mA) and nonvolatile resistive switching (RS) behavior at high CC (>0.5 mA). Additionally, Ag/Au-fibril hybrids/Pt memristors exhibit higher performance, i.e., high on-off ratio (>10^7^), low switching voltage (<0.8 V), reliable cycling, and retention characteristics. The conductive mechanism based on the SCLC model was explored and discussed in detail. Furthermore, the synaptic functions were emulated by our devices, such as EPSC, PPF, and the transition from STP to LTP. Finally, the logic cells of the AND and OR logic gate were simulated by Verilog-A mode in virtuoso, displaying the versatility of as-constructed biocompatible memristive devices. This work opens a door for amyloid-based materials or protein-assembled materials with electroactivity as the potential active layer in the construction of biocompatible memristive devices, which could be used well in brain-inspired applications and Boolean logic computing.

## Figures and Tables

**Table 1:** Comparison of other kinds of hybrid memristors.

Ref	Device Structure	Switching Voltage	Switching Mode	Switching Ratio
[36]	Al/PEDOT:PSS ^1^/ITO	V_SET_ < 1 V, V_RESET_ > −1.5 V	RS	N/A
[37]	Ag/DNA-CTMA ^2^:Ag./ITO	V_SET_ < 3 V	RS	>10^2^
[38]	Ti/Milk-Ta_2_O_5_/Pt	V_SET_ < 3 V, V_RESET_ > −1 V	RS	>48
[39]	Al/CDs ^3^-silk/ITO	V_SET_ < 3 V, V_RESET_ > −1 V	RS	>10^5^
[40]	Al/soybean:MWCNT ^4^/ITO	V_SET_ > −2 V, V_RESET_ < 4 V	RS	>10^4^
[41]	Mg/tyrosine-rich peptide/Mg/Au	V_SET_ < 4 V, V_RESET_ > −3 V	RS	>10^4^
**This Work**	**Ag/Au-fibrils hybrids/Pt**	**V_SET_ < 0.8 V,** **V_RESET_ > −0.7 V**	**TS/RS**	**>10^7^**

^1^ PEDOT:PSS: Poly(3,4-ethylenedioxythiophene)-poly(styrenesulfonate). ^2^ CTMA: cetyltrimethylammonium. ^3^ CDs: carbon dots. ^4^ MWCNT: multiwalled carbon nanotube.

## Data Availability

The data presented in this study are available on request from the corresponding author.

## Supplementary Materials

The following supporting information can be downloaded at: https://www.mdpi.com/article/10.3390/ma16051884/s1, Figure S1: Characterizations of hIAPP20–29 fibrils; Figure S2: Characterizations of the Au-fibrils nanocomposites; Figure S3: Characterizations of amyloid fibrils based memristive devices. Figure S4: (a) The cumulative distributions of the VSET and VRESET in 30 I-V cycles sweeping. (b) The average set voltage of 10 cycles for 18 devices.
