# Supplementary material for: Amyloid–Gold Nanoparticle Hybrids for Biocompatible Memristive Devices
Source: Materials (Basel). 2023 Feb 24;16(5):1884. doi: 10.3390/ma16051884 (PMC10004345; doi:10.3390/ma16051884)
Supplement: Supplementary file 1 [file materials-16-01884-s001.zip › materials-2211025-supplementary.pdf]

Amyloid–Gold Nanoparticle Hybrids for Biocompatible Memristive Devices

Aoze Han [1,†], Liwei Zhang [2,†], Miaocheng Zhang [1,3], Cheng Liu [3], Rongrong Wu [2], Yixin Wei [1], Ronghui Dan [1], Xingyu Chen [1], Ertao Hu [3], Yerong Zhang [3], Yi Tong [1,*], Lei Liu [2,*]

[1] College of Integrated Circuit Science and Engineering, Nanjing University of Posts and Telecommunications, Nanjing, 210023, China
[2] Institute for Advanced Materials, Jiangsu University, Zhenjiang, 212013, China
[3] College of Electronic and Optical Engineering & College of Flexible Electronics (Future Technology), Nanjing University of Posts and Telecommunications, Nanjing, 210023, China
* Correspondence: tongyi@njupt.edu.cn (Y.T.); liul@ujs.edu.cn (L.L.)
† These authors contributed equally to this work.

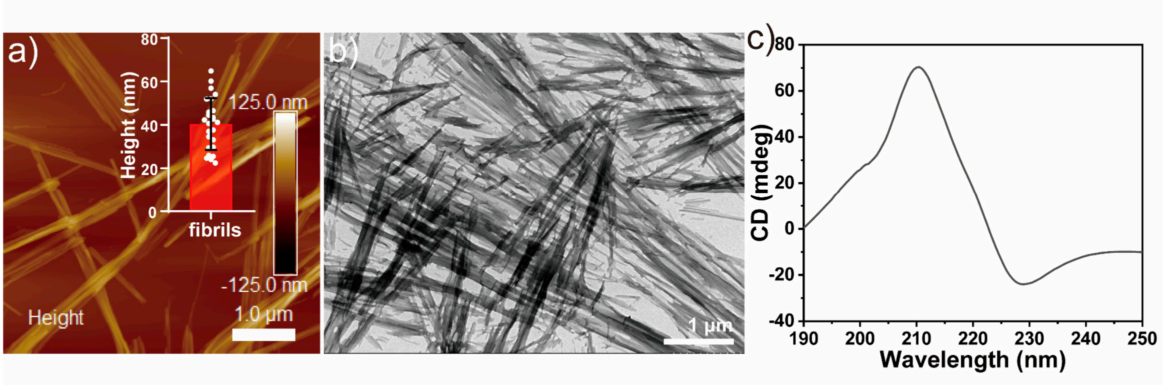

Figure S1. Characterizations of hIAPP$_{20\text{-}29}$ fibrils. (**a**) The AFM image of hIAPP$_{20\text{-}29}$ fibrils, the inserts are the corresponding fibrils size distribution; (**b**) The TEM image of hIAPP$_{20\text{-}29}$ fibrils; (**c**) Circular dichroism (CD) spectra of hIAPP$_{20\text{-}29}$ fibrils in Milli-Q water.

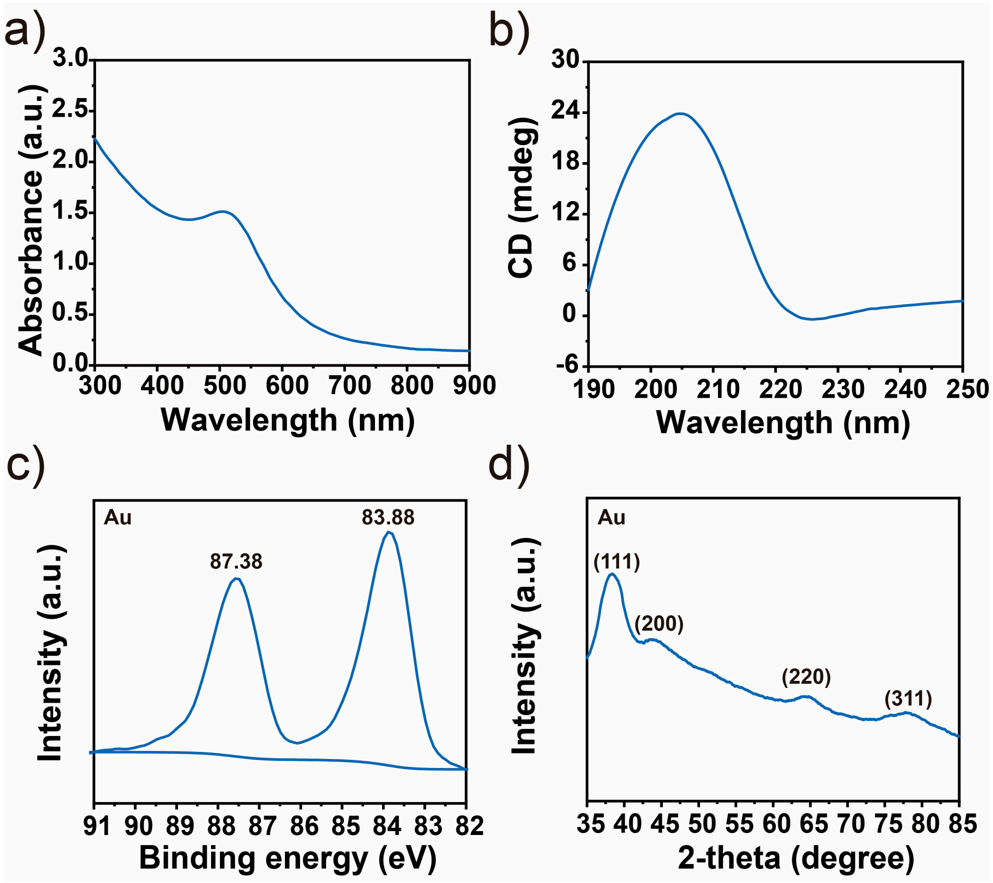

Figure S2. Characterizations of the Au-fibrils nanocomposites. (**a**) UV–vis spectrum of the Au-fibrils nanocomposites; (**b**) Circular dichroism (CD) spectra of the Au-fibrils nanocomposites in Milli-Q water; (**c**) XPS spectra of the Au-fibrils nanocomposites. (**d**) XRD spectra of the Au-fibrils nanocomposites.

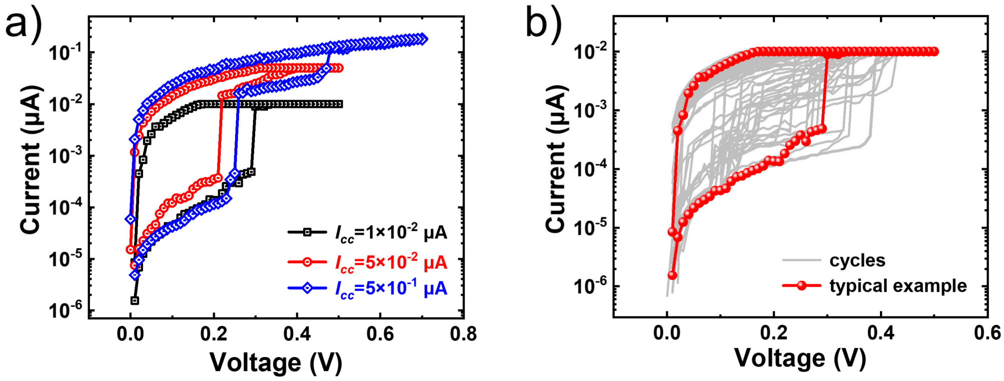

Figure S3. Characterizations of amyloid fibrils based memristive devices. (**a**) The current–voltage (I–V) characteristics of SET process under different current compliances set from 0.01 μA to 0.5 μA; (**b**) Threshold switching I–V characteristic of $hIAPP_{20-29}$ based memristor devices.

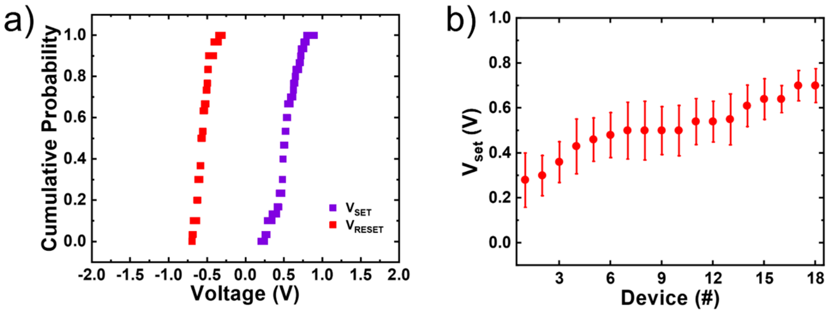

Figure S4. (**a**) The cumulative distributions of the V_{SET} and V_{RESET} in 30 I-V cycles sweeping. (b) The average set voltage of 10 cycles for 18 devices.
